# A machine learning approach for understanding the metabolomics response of children with autism spectrum disorder to medical cannabis treatment

**DOI:** 10.1038/s41598-023-40073-0

**Published:** 2023-08-22

**Authors:** Jean-Christophe Quillet, Michael Siani-Rose, Robert McKee, Bonni Goldstein, Myiesha Taylor, Itzhak Kurek

**Affiliations:** Cannformatics, Inc., 3859 Cesar Chavez St, San Francisco, CA 94131 USA

**Keywords:** Metabolomics, Machine learning, Biomarkers

## Abstract

Autism spectrum disorder (ASD) is a neurodevelopmental condition impacting behavior, communication, social interaction and learning abilities. Medical cannabis (MC) treatment can reduce clinical symptoms in individuals with ASD. Cannabis-responsive biomarkers are metabolites found in saliva that change in response to MC treatment. Previously we showed levels of these biomarkers in children with ASD successfully treated with MC shift towards the physiological levels detected in typically developing (TD) children, and potentially can quantify the impact. Here, we tested for the first time the capabilities of machine learning techniques applied to our dynamic, high-resolution and rich feature dataset of cannabis-responsive biomarkers from a limited number of children with ASD before and after MC treatment and a TD group to identify: (1) biomarkers distinguishing ASD and TD groups; (2) non-cannabinoid plant molecules with synergistic effects; and (3) biomarkers associated with specific cannabinoids. We found: (1) lysophosphatidylethanolamine can distinguish between ASD and TD groups; (2) novel phytochemicals contribute to the therapeutic effects of MC treatment by inhibition of acetylcholinesterase; and (3) THC- and CBD-associated cannabis-responsive biomarkers are two distinct groups, while CBG is associated with some biomarkers from both groups.

## Introduction

Autism spectrum disorder (ASD) is a set of heterogeneous neurodevelopmental conditions that affect social interaction and communication with defined stereotyped patterns of behavior^1^. It is a lifelong condition with onset as early as the first or second trimester that is often co-occurring with intellectual disabilities, psychiatric conditions, neuro-inflammation, and/or gastrointestinal disorders^2–5^.

Diagnosis and evaluation of treatment efficacy are challenging due to the clinical phenotypic heterogeneity of ASD and currently rely solely on subjective evaluation by developmental pediatricians, neurologists, or psychologists. As such, observational survey tool scores are not comparable among patients, and do not provide information regarding the underlying pathophysiology of ASD. Since the onset of ASD is triggered by both genetic and environmental factors via a cascade of biochemical events that leads to pleiotropic metabolic abnormalities with high variability among the individuals, it is challenging to identify ASD biomarkers^6^. The fact that the risk of having a second child with ASD is 25-fold higher for families that already have a child with ASD as compared to families with a typically-developing (TD) child strongly suggests the involvement of genetic factors^7^. However, genetic biomarkers associated specifically with ASD have not been identified or routinely used for screening. Abnormal levels of proteins and metabolites related to oxidative stress, inflammation, mitochondrial dysfunction and immune dysregulation have been identified and characterized in ASD in the last two decades^8^. Nonetheless, the high metabolic variability among individuals with ASD and comorbidity associated with other disorders have limited the development of reliable proteomic and metabolic biomarkers for diagnosis and treatment evaluation.

Machine learning (ML) is a subfield of artificial intelligence (AI) in which a variety of statistical and computational methods are applied to large and complex datasets in order to develop and/or fit predictive models by imitating human pattern-recognition processes^9^. ML requires a training dataset consisting of data points each considered as a single observation from an experiment that is described by number of features. A sufficient number of trained features permits the development of a model that predicts the output. ML techniques have been successfully applied to metabolomics studies to identify the following: the metabolic signature of severe COVID-19 cases, the taxonomy of human gut microbiota, metabolic changes in human pregnancy, influenza infection, renal cell carcinoma (88% accuracy), diabetic kidney disease, head and neck paragangliomas (99.2% accuracy), early-stage bladder cancer (up to 95% accuracy) and the metabolomics signatures of major depressive disorder subtypes^10^. Chen et al.^11^ combined GC/MS-based untargeted urine metabolomics of samples collected from a group of children with ASD and a TD control group with the XGBoost algorithm to identify 20 potential metabolic biomarkers to distinguish between the groups, which were mapped to a variety of metabolic pathways.

MC treatment is emerging as a promising solution for treatment of children with ASD, especially in the absence of approved medications that fail to treat the core symptoms of ASD. Improvements in the social communication skills of children and adolescents with ASD treated with CBD-rich cannabis were recently reported by Hacohen et al.^12^, and improvement in behavioral outbursts, anxiety, and communication were reported for children with ASD treated with low tetrahydrocannabinol (THC) and high cannabidiol (CBD) formulations^13^. These studies used subjective evaluations, limiting the ability to quantify the impact of MC treatment. With the increase of MC treatment of children and adolescents with ASD, there is a growing need for objective, quantified data to determine the effectiveness, safety, mechanism of action and cellular targets of cannabinoids.

We have recently reported on a new pharmacometabolomics approach in which the levels of metabolites, specifically cannabis-responsive biomarkers, in children with ASD being treated with MC shifted toward the physiologic levels detected in untreated typically developing (TD) children^14,15^. In these investigations, we identified a possible link between cannabis-responsive biomarkers and mitochondrial dysfunction, alterations in neurotransmitters, abnormal neuronal development, neuroinflammation, bioenergy and oxidative stress. Importantly, the cannabis-responsive biomarkers quantified the impact of MC treatment on the children with ASD, shedding light on the underlying pathophysiology of ASD and indicating a possible mechanism of action (MOA) of cannabinoids.

In our previous studies^14,15^ we used hard-coded algorithms to identify and rank biomarkers that solely shift toward physiological levels. Here we explore the potential of the cannabis-responsive biomarker database, which contains a large number of metabolites detected in a limited number of children with ASD, regardless of the physiological outcomes, and TD controls, for ML applications primarily yielding new biomarker candidates overlooked in previous studies. We trained Gradient Boosting models to: (1) distinguish individuals with ASD from the control group before and after MC treatment; (2) identify non-cannabinoid plant molecules (phytochemicals) with medicinal benefits that contribute to the synergistic effect known as the entourage effect^16^; (3) distinguish specific THC-, CBD- and cannabigerol (CBG)-responsive biomarkers; and (4) provide insights on the specific impact of cannabinoids on imbalanced metabolic pathways in children with ASD. Gradient Boosting-based ML models perform well with high dimensional data where the number of features exceeds the number of samples, and provide us with a straightforward method to rank these features, the metabolites, according to their relative contribution to the prediction for each task.

The preliminary results presented in this study demonstrate the potential of the cannabis-responsive biomarker database in conjunction with ML applications to provide insight into the pharmacokinetics, pharmacodynamics and MOA of cannabinoids and on targets in endocannabinoid system (ECS)-related disorders, and to identify new *Cannabis* phytoconstituents with potential therapeutic roles.

## Results

### Potential cannabis-responsive biomarker database characteristics

Fifteen children (average age 9.4 years) participated in the ASD group and 10 children with a similar age distribution (average age 9.3 years) participated in the TD untreated control group as previously described in detail^14,15^. Within the ASD group 11 children exhibited severe range, 2 children exhibited moderate range and 2 children exhibited a mild range of social impairment associated with ASD as reported by parent ratings (SRS-2), as described in detail by Siani-Rose et al.^14^ (Suppl 2).

We applied ML applications to source data for each child containing the absolute values of 645 metabolites detected in the saliva collected from study participants by dual scan capillary electrophoresis time-of-flight-mass spectrometry (CE–TOF–MS) and rapid resolution liquid chromatography-time-of-flight-mass spectrometry (RRLC–TOF–MS)^14,15^. The samples were designated in children with ASD before MC treatment as PRE and approximately 90 min after MC treatment as PEAK, and a single sample from children in the untreated TD control group as (TD). For some children with ASD, we also collected samples designated Post-1 and Post-2 approximately 180 and 270 min after MC treatment, respectively. This data was supplemented with the numerical data shown in Table 1, in which the doses of the major cannabinoids used in the MC treatment were grouped onto scales ranging from 0–3 (THC), 0–4 (CBD) and 0–2 (CBG); and parent behavioral rating surveys at PRE and PEAK were grouped on a scale of 0–3. THC was part of the treatment for 80% of the children, CBD was part of the treatment for 67% of the children and CBG was part of the treatment for 33% of the children. All the children were taking THC or CBD, with 47% taking both THC and CBD, and 7% taking THC, CBD and CBG. This ranking included all the practical combinations of THC, CBD and CBG with sufficient statistical power. According to behavioral rating surveys, parents of the ASD group reported full and partial improvement after MC treatment (PEAK vs PRE) in 80% of children.

**Table 1:**
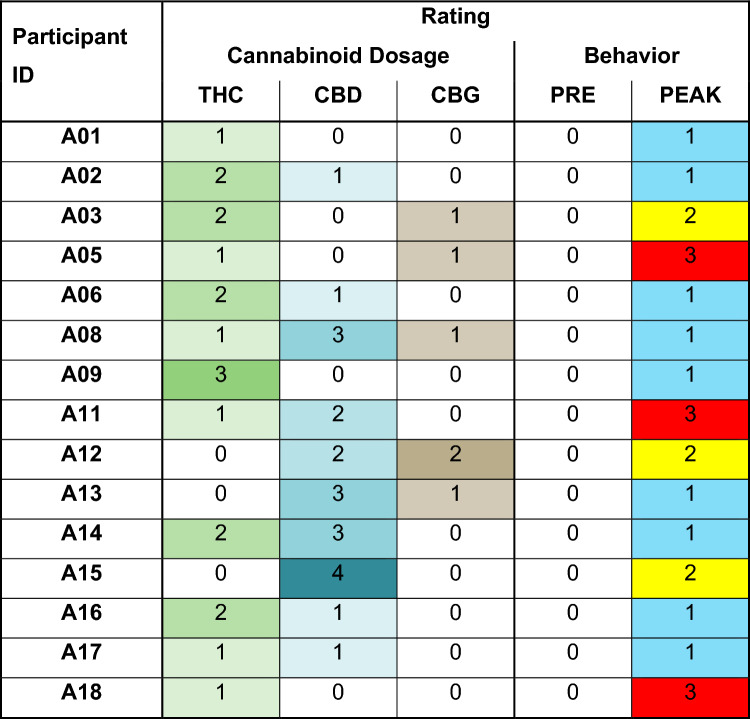
Numerical scales of major cannabinoids and behavioral rating surveys used for datasets of children with ASD.

Cannabinoid dosages were grouped according to the following concentrations: THC: (0) No; (1) 0.05–5.00 mg; (2) 5.05–15.00 mg; and (3) > 15.05 mg. CBD: (0) No; (1) 1–0 mg; (2) 31-84 mg; (3) 85–100 mg; and (4) > 100 mg. CBG: (0) No; (1) 1–49 mg; (2) > 50 mg. Color intensity represents the group ranking, with no color at 0. Behavior ranking at PRE and PEAK time points were numbered and color coded as follows: (1, blue) improved; (2, yellow) partially improved; and (3, red) worsened.

### Differentially-expressed potential cannabis-responsive biomarkers distinguish categories of children with ASD

**Figure 1:**
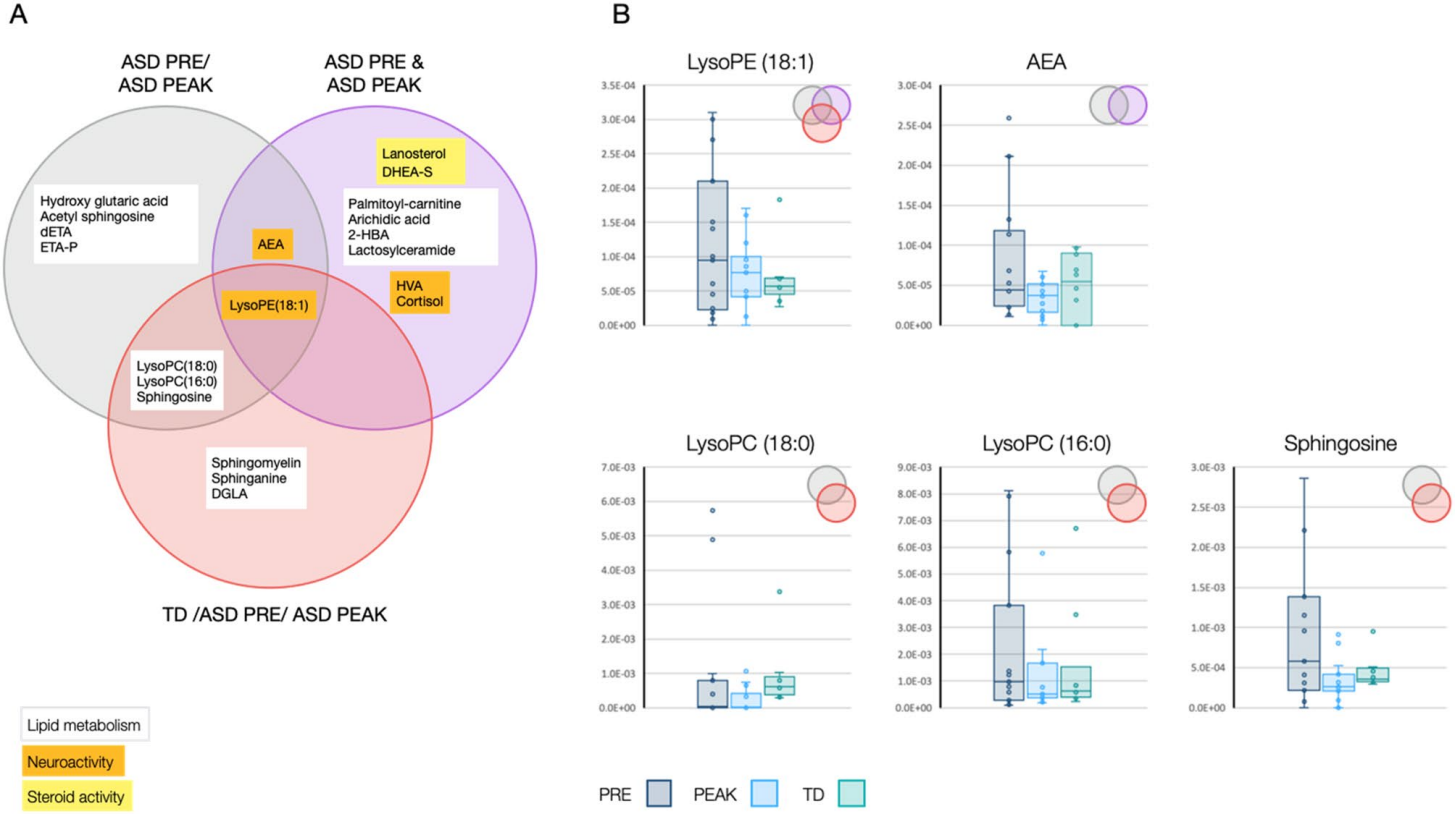
Identification of potential ASD cannabis-responsive biomarkers that distinguish categories of patients. (**A**) Venn diagram illustrating the unique and overlapping differentially-expressed cannabis-responsive biomarkers found in the categories of patients with ASD PRE/ASD PEAK, ASD PRE and ASD PEAK and TD/ASD PRE/ASD PEAK. The biomarker roles (lipid metabolism, neuroactivity and steroid activity) are color coded (white, orange and yellow, respectively). (**B**) Levels of potential cannabis-responsive biomarkers found in children with ASD at PRE (blue) and PEAK (light blue), and TD group (green) in the overlapping categories described in (**A**). Each box plot horizontally enclosed by the lower and upper quartiles and median (solid horizontal line within the box) is indicated. The overlapping categories are indicated in the upper right corner.

Using ML Gradient Boosting for Multi-Class classification of the metabolomics samples and the resulting importance ranking of the features for model prediction, we have generated 3 categories of potential biomarkers described below and in Fig. 1A.

(1) ASD PRE/ASD PEAK

Nine potential cannabis-responsive biomarkers were identified as candidates for distinguishing before MC treatment (PRE) or after MC treatment (PEAK), including the neuroactive compounds anandamide (AEA), lysophosphatidylethanolamine (LysoPE18:1), the neurodegenerative-associated lipids lysophosphatidylcholine (LysoPC18:0 and LysoPC16:0) and sphingosine, and the lipids/lipid pathway compounds hydroxy glutaric acid, acetyl sphingosine, diethanolamine (dETA), and ethanolamine phosphate (ETA-P).

(2) ASD PRE and ASD PEAK

Ten potential cannabis-responsive biomarkers were identified as candidates for distinguishing all children with ASD treatment where both PRE and PEAK are combined in a single string including the neuroactive compounds AEA, LysoPE(18:1), homovanilic acid (HVA), cortisol; the lipids palmitoyl-carnitine, arichidic acid, 2-hydroxy butyric acid (2-HBA), lactosyl ceramide; and the steroid/derivative lanosterol and Dehydroisoandrosterone 3-sulfate (DHEA-S).

(3) TD/ASD PRE/ASD PEAK

Seven potential cannabis-responsive biomarkers were identified as candidates for distinguishing the TD control group and the ASD children at PRE and PEAK, including the neuroactive LysoPE(18:1), sphingosine, the neurodegenerative-associated lipids LysoPC18:0 and LysoPC16:0, sphingomyelin, sphinganine and eicosatrienoic acid (DGLA).

LysoPE (18:1) was the only potential cannabis-responsive biomarker for distinguishing all 3 categories, as indicated by the Venn diagram (Fig. 1A). The overall high LysoPE (18:1) levels and high sample variability (Fig. 1B) found in children with ASD at PRE (blue) decreased at PEAK (light blue) but did not reach the low levels and low variability detected in the TD group (green). This expression pattern fits the criteria of a biomarker that can be used to distinguish all three categories, namely ASD PRE/ASD PEAK, ASD PRE and ASD PEAK and TD/ASD PRE/ASD PEAK.

The endocannabinoid AEA, which functions as a neurotransmitter and is produced and released “on demand”^14^, was found to overlap the categories ASD PRE/ASD PEAK and ASD PRE and ASD PEAK, with the overall lowest levels and lowest variability in children with ASD at PEAK.

The potential lipid-based cannabis-responsive biomarkers LysoPC (18:0), LysoPC (16:0) and sphingosine overlapped in the ability to distinguish the ASD PRE/ASD PEAK and TD /ASD PRE/ASD PEAK categories by reducing the high levels and high sample variability at PRE to low levels and low sample variability at PEAK, reaching a range and sample variability closer to the levels obtained in the TD control group.

### Differentially-expressed plant metabolites distinguish categories of patients

**Figure 2:**
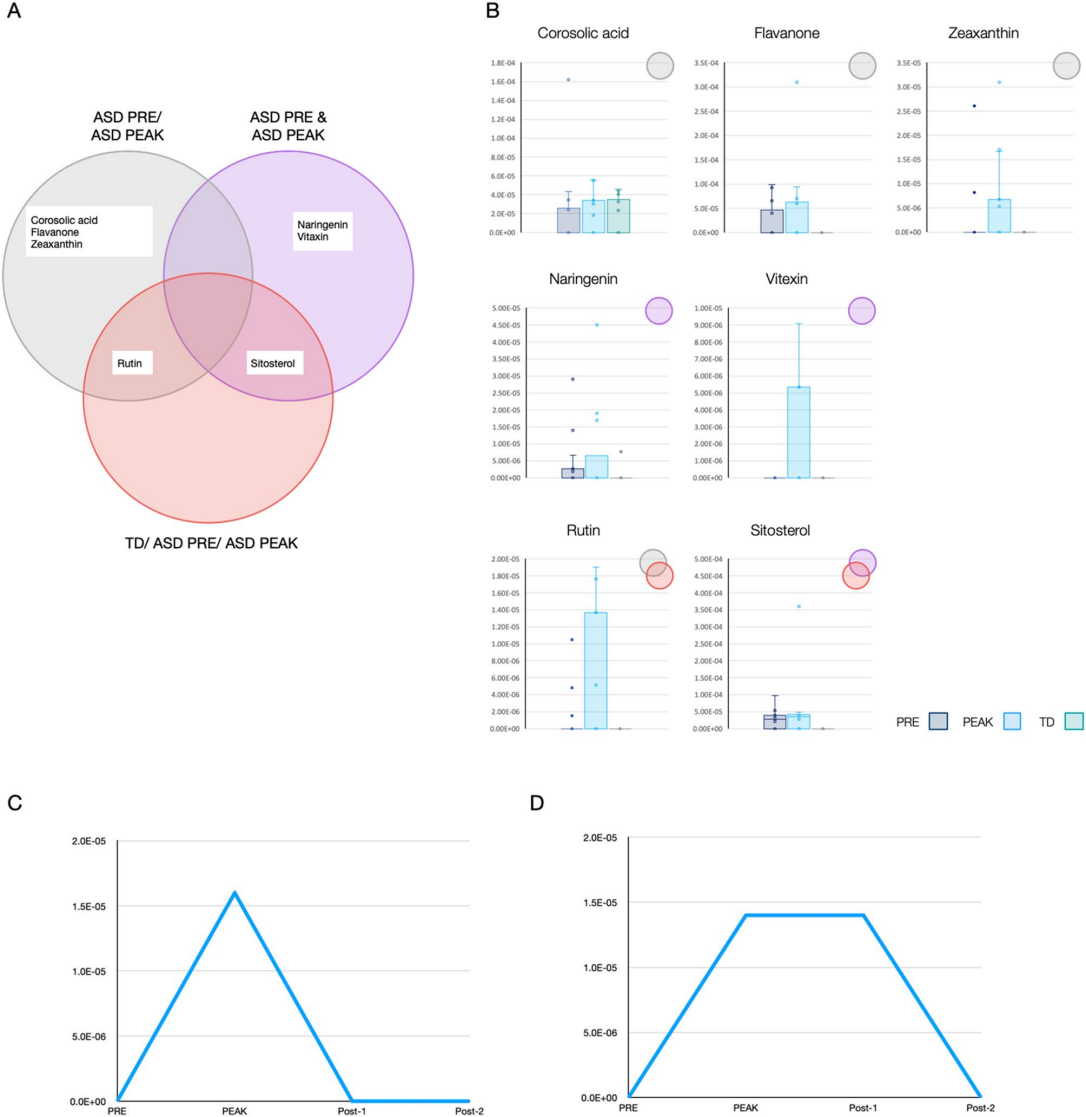
Identification of plant non-cannabinoid secondary metabolites (dietary phytochemical) that distinguish categories of patients. (**A**) Venn diagram illustrating the unique and overlapping dietary phytochemicals found in the categories of patients ASD PRE/ASD PEAK, ASD PRE and ASD PEAK and TD/ASD PRE/ASD PEAK. The dietary phytochemical functions (lipid, neuroactive and steroid) are color coded (white, orange and yellow, respectively). (**B**) Levels of dietary phytochemicals found in children with ASD at PRE (blue) and PEAK (light blue), and TD group (green) in the overlapping categories described in (**A**). Each box plot horizontally enclosed by the lower and upper quartiles and median (solid horizontal line within the box) is indicated. The overlapping categories are indicated in the upper right corner. (**C**) Time dependent levels of vitexin (apigenin 8-glucoside) detected at time points PRE (10 min before MC treatment), PEAK, Post-1 and Post-2 (90, 180 and 270 min after MC treatment, respectively) in child ID A18. (**D**) Time dependent levels of rutin (quercetin 3-rutinoside) detected at time points described in (**C**) in child ID A16.

The approach described above successfully identified plant metabolites for distinguishing categories of children with ASD (Fig. 2A). Seven dietary phytochemicals including flavone, rutin (quercetin-3-rutinoside), vitexin (Apigenin 8-glucoside), naringenin, zeaxanthin, corosolic acid and sitosterol were detected in children with ASD at PEAK (Fig. 2B). Sitosterol was the most abundant dietary phytochemical detected in the saliva of 10 children with ASD. Rutin, vitexin and naringenin were less abundant and were each detected in only 5, 4 and 4 children (respectively). Corosolic acid was the only dietary phytochemical detected in both the ASD and TD control group and exhibited a slightly increased amount in PEAK vs PRE toward the levels seen in TD subjects.

As indicated in the Venn diagram in Fig. 2A, rutin overlapped the ASD PRE/ASD PEAK and TD/ASD PRE/ASD PEAK categories, and sitosterol overlapped the ASD PRE and ASD PEAK and TD/ASD PRE/ASD PEAK. Time dependent detection of vitexin in saliva samples of child A18 and rutin in saliva samples of child A16 (Fig. 2C,D respectively) indicated a different bioavailability pattern in which vitexin degrades faster than rutin. Therefore, the detected levels of rutin in the saliva of children with ASD at PRE could be a result of previous treatment.

### The impact of major cannabinoids on differentially-expressed potential cannabis-responsive biomarkers distinguishing categories of children with ASD

**Figure 3:**
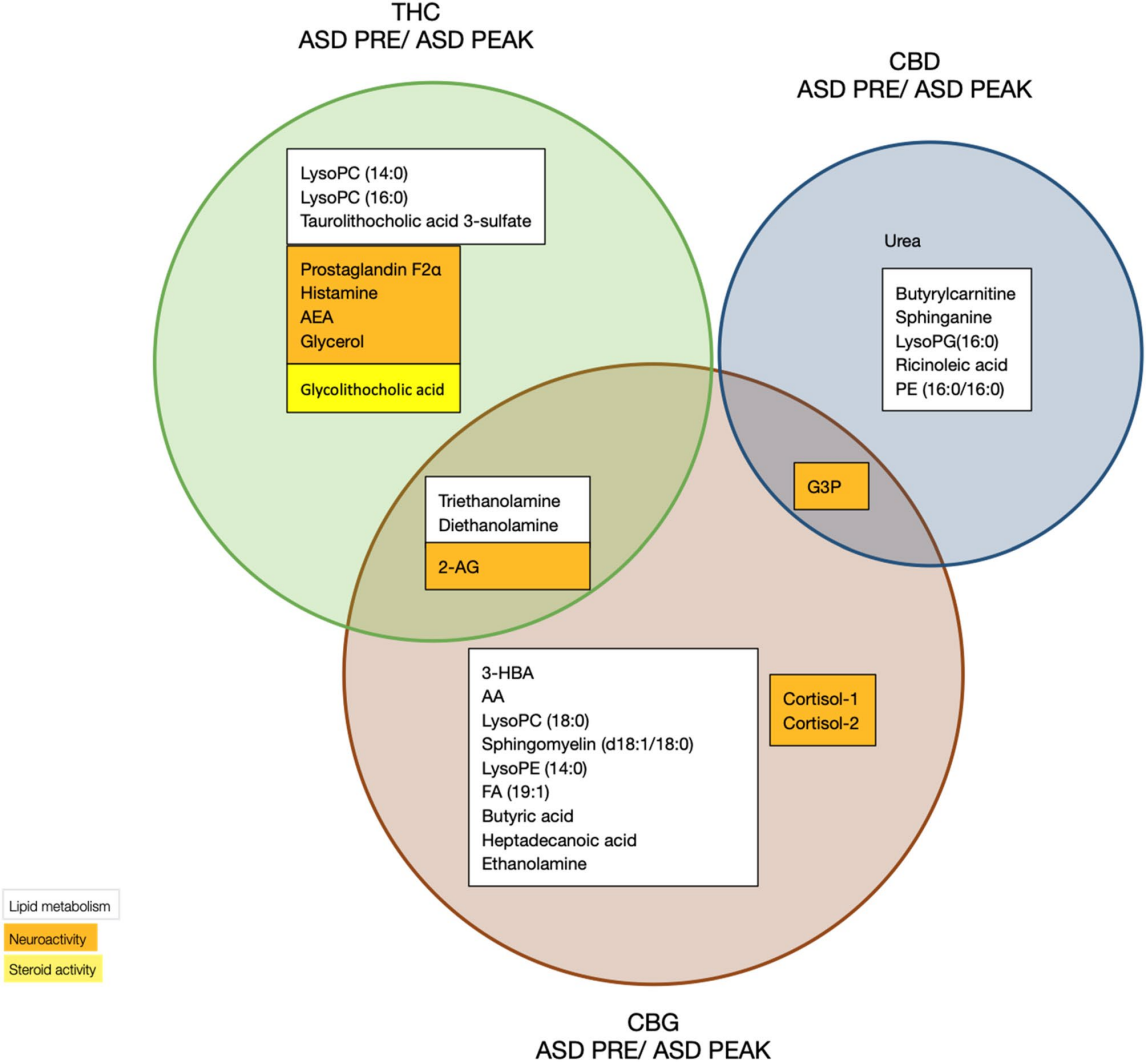
Identification of potential THC-, CBD- and CBG-responsive biomarkers that distinguish patients with ASD at PRE vs PEAK. Venn diagram illustrating the unique and overlapping cannabis-responsive biomarkers that respond (PRE/PEAK) to THC, CBD and CBG treatment. The biomarker functions (lipid metabolism, neuroactivity and steroid activity) are color coded (white, orange and yellow, respectively).

Using ML Gradient Boosting for Multi-Class classification of the metabolomics samples and the resulting importance ranking of the features, we have analyzed the specific contribution of THC, CBD and CBG found in the MC treatment described in Table 1 in a data set of 645 metabolites detected in 30 saliva samples from 15 children with ASD at PRE and PEAK time points. As shown in Fig. 3, THC was associated with the response of 11 potential cannabis-responsive biomarkers representing lipids, neuroactive molecules and steroids (5, 5 and 1 respectively), including the endocannabinoids AEA and 2-arachidonoylglycerol (2-AG). CBD was associated with 7 potential cannabis-responsive biomarkers with roles in lipid metabolism, neuroactivity and protein metabolism (5, 1, and 1 respectively). The 11 THC potential cannabis-responsive biomarkers did not overlap with the 7 CBD potential cannabis-responsive biomarkers. CBG was associated in the response of 15 potential cannabis-responsive biomarkers with roles in lipid metabolism and neuroactivity (11, and 4 respectively). Three CBG potential cannabis-responsive biomarkers overlapped with THC, including the endocannabinoid 2-AG, di- ethanolamine (dETA) and tri-ethanolamine (tETA); and one CBG potential cannabis-responsive biomarker, glycerol 3-phosphate (G3P), overlapped with CBD.

## Discussion

Metabolic biomarkers are dynamic components of the omics disciplines (genomics, transcriptomics, proteomics, and metabolomics) closest to phenotype that can quantify physiological changes^10^. These biomarkers are being successfully used for patient stratification, diagnosis, monitoring, pharmacodynamic/response, and predictive tools^17^. We have recently reported on salivary cannabis-responsive biomarkers, metabolites that objectively measure the response to MC treatment in children with ASD and indicating the impact in comparison to targeted values determined in a TD population^14,15^. Cannabis-responsive biomarkers that provide a high-resolution snapshot of cannabinoid-dependent metabolic changes are the closest quantifiable step to the phenotypic evaluation currently used in children with ASD. Using insights from Gradient Boosting-based ML predictors with the limitations of a small patient dataset, we were able to successfully utilize the complex cross-features (metabolites) and determine the possible impact of MC treatment on the role of known and annotated metabolic pathways in children with ASD. In addition, we were able to associate new plant-based non-cannabinoid metabolites with the therapeutic impact in our ASD treatment group, which has not been previously reported.

### Biomarkers for distinguishing categories of children with ASD based on response to MC treatment

In our previous studies we used different algorithms on the same dataset in order to identify cannabis-responsive biomarkers using a screening and sorting methodology for beneficial outcomes^14,15^. The previous algorithms screened for metabolites that meet the following therapeutic importance criteria, namely: (1) changes in response to cannabis in 60% or more children with ASD; (2) levels after MC treatment (PEAK) significantly different from PRE; and (3) the highest number of children in which the levels shifted toward the physiological range determined as 2 standard deviations (SDEVs) from the average found in the TD group. In this study we applied ML tools to distinguish between categories of children with ASD at PRE, PEAK and the TD control focusing on unique metabolites that differentiate categories (i.e. PRE, PEAK, TD) regardless of the positive or negative outcome.

Within the TD/ASD PRE/ASD PEAK category, the ML predictors can include biomarkers that were identified in the previous study that moved toward the TD range (therapeutically beneficial) and/or new biomarkers that moved away from the TD range (therapeutically harmful) after MC treatment. We identified 7 metabolites in the TD /ASD PRE/ASD PEAK category that have been previously characterized as potential cannabis-responsive biomarkers (Siani-Rose et al.^14^, Suppl 1) with roles in lipid metabolism^15^ and linked to ASD. Moreover, we could not find any evidence of negative impact of MC, namely a metabolite in which the sample distribution in the PRE group was similar to the TD group and subsequently increased (shifted away from TD range) at PEAK (Fig. 1B). This could be explained by the selection of children for the ASD group that were successfully treated with MC under physician supervision.

Among the three categories described in Fig. 1, the neuroactive lipid-based cannabis-responsive biomarker LysoPE(18:1) was the only distinguishing factor in all 3 categories (ASD PRE/ASD PEAK, ASD PRE and ASD PEAK and TD/ASD PRE/ASD PEAK), which may indicate a partial impact of MC treatment on important ASD underlying conditions. Abnormal levels of LysoPE(18:1) detected in the hippocampus of rats were previously suggested as an indicator of postischemic cognitive impairment^18^. The endocannabinoid AEA was the only metabolite distinguishing factor in the categories ASD PRE/ASD PEAK and ASD PRE and ASD PEAK but not the TD group. AEA responds to MC similarly to all the other metabolites and overlapped at least 2 categories described in Fig. 1B by decreasing the large sample distribution range detected at PRE to the small distribution range obtained in the TD group. This tighter distribution at PEAK suggests down-regulation of the endocannabinoid AEA in response to cannabinoid treatment, potentially suggesting improved ECS tone. Our observations further support Di Marzo et al.^19^ in which repeated treatment with THC reduced the AEA biosynthetic precursor N-arachidonoylphosphatidylethanolamine (NArPE) content and signaling in the striatum of rats.

Within the unique metabolites obtained specifically in the ASD PRE/ASD PEAK or the ASD PRE and ASD PEAK category, only palmitoyl-carnitine, HBA, HVA and cortisol met the cannabis-responsive biomarker criteria previously reported (Siani-Rose et al.^14^, Supp 1), while all the others were identified in our previous study but did not meet the first criteria, namely present in 60% or more children^14^. For example, DHEA-S associated with aggression in psychiatric disorders^20^ was detected at high levels only in young adolescents (11–12 years old boys).

### Non-cannabinoid plant phytochemicals with potential entourage effects

The ability to identify metabolites distinguishing TD/ASD PRE/ASD PEAK allowed us to associate known plant phytochemicals with successful MC treatment observed by parental evaluation and previously reported in Siani-Rose et al.^14^ Supp 3. Phytochemicals are secondary plant metabolites such as polyphenols (e.g. flavonoids), terpenoids (e.g.carotenoids) and phytosterols (e.g. sterols) with medicinal properties such as anti-inflammatory, antioxidant and antibacterial^21^. The cannabis monoterpenes such as limonene, myrcene, and linalool, that share the common C-10 precursor geranyl diphosphate (GPP) with CBGA, are considered to provide a phytocannabinoid-terpenoid synergistic effect known as the entourage effect^16^. In this study, we have identified 7 plant-based molecules, all dietary phytochemicals from 3 groups all previously reported to inhibit acetylcholinesterase: polyphenols (flavone^22^, rutin^23^, vitexin^24^ and naringenin^25^), terpenoids (zeaxanthin^26^ and corosolic acid^27^) and phytosterols (sitosterol^27^) (Fig. 2A,B). Since 6 phytochemicals (flavone, rutin, vitexin, naringenin, zeaxanthin and sitosterol) were not detected in the TD group and exhibited increased levels at PEAK, it is possible to characterize this acetylcholinesterase (AChE) inhibition activity as an entourage effect. In this respect, AChE inhibition by the drug galantamine was previously reported to effectively reduce irritability and lethargy/social withdrawal in children with ASD^28^.

### Biomarkers for distinguishing association with major cannabinoids in children with ASD based on response to MC treatment

Cannabis-responsive biomarkers specifically categorized by THC, CBD and CBG can indicate the metabolic pathways that are affected by cannabinoids in children with ASD (Fig. 3). This is a preliminary step in understanding the MOA of cannabinoids and a path to personalized MC treatment. We found that THC interacts with the retrograde cannabinoid signaling pathway (KEGG map 04723; Kyoto Encyclopedia of Genes and Genomes, https://www.kegg.jp) by changing the levels of 2-AG and AEA. THC therefore plays an important role in signaling across the synaptic cleft, and in the binding and activating CB1 receptor found in both the neural membranes at the synapse and in the mitochondrial membrane in the excitatory and inhibitory terminals^29^. CBG was found to affect the levels of 2-AG only, while CBD did not affect any of the endocannabinoids, suggesting a different MOA. Moreover, THC, CBD and CBG trigger changes in the glycerophospholipid metabolism pathway (KEGG map00564), and CBD and CBG affect sphingolipid metabolism (KEGG map00600). While THC changes the levels of lipid-, neuroactive- and steroid-based biomarkers, CBD mainly affected lipid-based biomarkers as suggested by Veilleux et al. via the extended ECS or the endocannabinoidome (eCBome)^30^. CBGA, the acidic form to CBG, is also the precursor to THCA and CBDA, which convert to THC and CBD via decarboxylation at elevated temperature. CBG was less specific and can possibly function as a “bridge” between THC and CBD based on overlapping biomarkers obtained in this study; this adds support to the findings by Nachnani et al.^31^, namely that “CBG seems to reside, pharmacologically, in between THC and CBD.”

### ML and expanding knowledge of metabolic pathways

**Figure 4:**
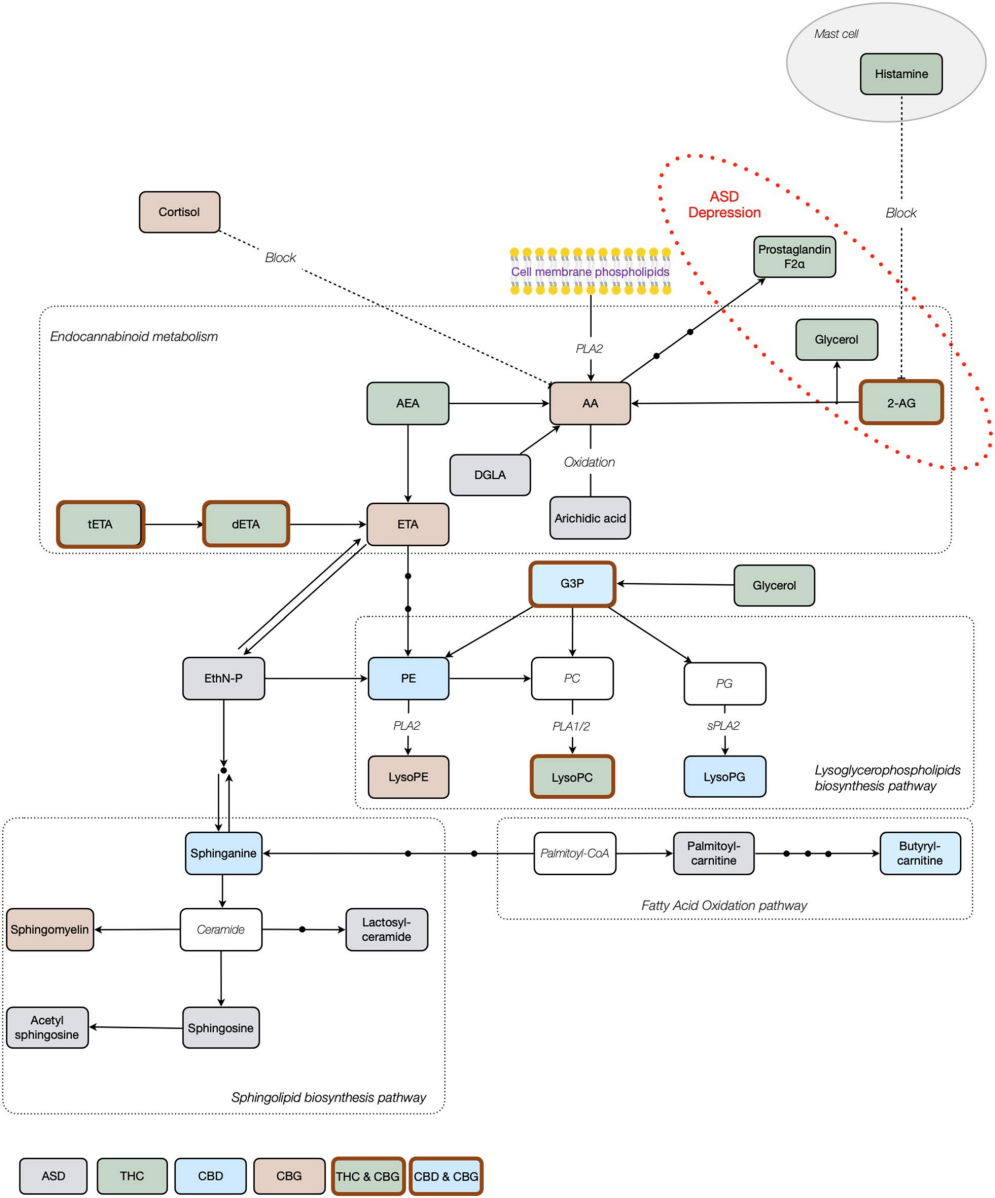
Simplified metabolic pathways associated with the differential expression of potential ASD cannabis-responsive biomarkers after THC, CBD and CBG treatment. Potential ASD cannabis-responsive biomarkers directly respond to THC (green), CBD (blue) and CBG (brown) found in the metabolic pathways of lysoglycerophospholipids, sphingolipid, fatty acid oxidation, anandamide and ethanolamine-phosphate (EthN-P), and their impact on ASD and depression, are indicated. Potential ASD cannabis-responsive biomarkers previously only identified and described in Siani-Rose (2021 and 2022) are in gray, with metabolites in white.

By taking together the ML data obtained in this study and the known metabolic pathways of endocannabinoids^32^, lysoglycerophospholipids^33^, sphingolipid^34^, and fatty acid oxidation^35^ in ASD^36^, markers of depression^37^ and pathways containing histamine^38^, we were able to assemble a preliminary simplified THC-, CBD- and CBG- responsive metabolic pathway in children with ASD (Fig. 4). We also introduced general cannabis-responsive biomarkers previously described in Siani-Rose^14,15^ to this simplified metabolic pathway. The endocannabinoid metabolic pathway was strongly linked with THC, including with both AEA and 2-AG. CBG was associated with 2-AG, arachidonic acid (AA) and ethanol amine (ETA), while tETA and diETA were associated with both THC and CBG.

Studies suggested that ASD^36^ and Alzheimer’s Disease^40^ are linked to neuroinflammation-associated increased activity of the brain phospholipase A2 (PLA2) that specifically converts cell membrane phospholipids to arachidonic acid (AA). Similarly, Esvap and Ulgen^34^ reported increased PLA2 hydrolysis activity of phospholipids into AA and polyunsaturated free fatty acids (PUFA) in children with ASD. In this respect, the association of CBG with AA may provide an insight into its anti-inflammatory role by reducing AA levels in children with ASD (PRE/PEAK) possibly via PLA2. This CBG-associated AA was also linked with the THC-associated metabolic pathway that includes 2-AG, prostaglandin F2α and glycerol, all of which are involved in ASD^36^ and in depression^37^.

Lysoglycerophospholipids are hydrolyzed glycerophospholipidlipids involved in signaling and membrane biosynthesis. Our preliminary data showed that members of lysoglycerophospholipid pathways are possibly associated with THC (LysoPC), CBD (LysoPG) and CBG (LysoPE and LysoPC), all of which are products of the phospholipase A (PLA) enzymes sPLA2, PLA2 and PLA1 and 2, respectively^33^. A significant increase in PLA2 activity was previously reported by Bell et al.^39^ and by Qasem et al.^41^, who showed a decrease in the mean concentrations PE and PC, the substrates of LysoPE and LysoPC respectively. This is in agreement with our observations showing increased levels of LysoPE and LysoPC at PRE (Fig. 1) that were reduced in response to MC treatment containing THC, CBD and/or CBG. Qasem et al.^41^ suggested that inflammation increases PLA2 levels via mediators in a response to oxidative stress in children with ASD. It is possible that THC, CBD and/or CBG prevent inflammation and therefore reduce the levels of PLA2.

The sphingolipid biosynthesis pathway was associated with CBD (sphinganine) and CBG (sphingomyelin). Three lipids in the sphingolipid biosynthesis pathway, namely, sphingosine, lactosyl-ceramide and acetyl sphingosine, detected in high levels at PRE in children with ASD, were not associated with any cannabinoid (Fig. 1). While low/no detectable levels of ceramide were obtained in our study^14,15^, the high levels detected in its direct product sphingosine (Fig. 1B) and sphingomyelin (not shown) could be explained by the high production of ceramide that quickly converted to other derivatives including sphingosine and sphingomyelin. Esvap and Ulgen^34^, using transcriptomics data to develop an ASD-specific genome-scale metabolic model (GEM), have suggested that ceramide biosynthesis is induced by oxidative stress in children with ASD and quickly hydrolyzed. Our data support this hypothesis as MC treatment in general^14,15^ and CBD and CBG in this study were linked with reduced oxidative stress.

### ML and cannabis-responsive biomarker database

Developing cannabis-responsive biomarkers is the first step to objectively quantify the impact of MC treatment. It also presents an opportunity to better understand the relationships between these biomarkers and cannabinoids using ML applications, and to obtain insight into the MOA of active cannabinoids on the underlying conditions of ASD. Cannabis-responsive biomarkers provide a dynamic, high-resolution and rich feature dataset in several aspects, as shown in this study: (1) the broad range of detection levels, in which the highest detectable levels of the metabolite lactic acid were 19,000-fold higher than the oxidized glutathione acid levels on a molar basis; (2) the large number of features, i.e. the metabolites and potential biomarkers in each sample; (3) each child is a dynamic (before and after treatment) independent study; and (4) the ability to analyze large numbers of correlations, both linear and complex, among a large number of features and samples. These four aspects, which create a dynamic, high-resolution and rich feature dataset for ML applications, are the key to identifying new potential biomarkers, even with low-distinguishing factors or only in combination with any number of other biomarkers. This dataset also supports the personalized medicine approach, especially for MC treatment, as metabolic changes reflect both genetic and environmental factors, with samples before and after treatment, forming the basis of accurate ML predictions for therapy in the future.

## Conclusions

While further large sample size studies are needed to develop a large statistically-robust database, cannabis-responsive biomarkers combined with Gradient Boosting-based ML techniques can successfully personalize ECS-related MC therapy. It can also provide a metabolic snapshot in which MC treatment can be used as a probe to highlight ASD-related metabolic pathways by temporarily switching ASD pathophysiology to homeostasis. Additionally, our preliminary results suggest that ML applications can identify the specific MOA of cannabinoids and entourage effect of phytochemicals without the need to test each one separately.

### Limitations

Although the limitations to our pilot observational study were previously discussed in Siani-Rose et al.^14^ and Siani-Rose et al.^15^, we consider several additional limitations relevant to this study. First, the small sample size of the ASD group does not represent the full clinical phenotype heterogeneity found in the entire ASD population and thus we cannot suggest that MC treatment is relevant to all individuals with ASD. Second, each child was considered as single case treated with unique cannabinoid content and regimen. Thus, association of biomarkers with cannabinoid potency is limited to a range of concentrations arbitrarily determined and does not represent direct linear correlation. Third, each observational study was conducted on a single day and represents the child’s behavior a single time point that may not reflect the full range of behaviors which are influenced by environmental factors and can vary from day to day. Forth, the biomarker population is biased toward children successfully treated with MC. Fifth, the parents used a dropper to treat the children with prescribed off-the-shelf MC, which may introduce inaccuracy in the levels of dose reported. Sixth, the authors did not verify the cannabinoid content and potency of the MC treatment reported by the parents. Seventh, since the biomarkers were detected in saliva, some may not represent their relevant physiological role. Eighth, the small size of the data set combined with the much larger number of features (molecules) and heterogeneity of regimen is not optimal for the training, and then validation and testing of ML prediction models. A future study to generate a dataset from a larger cohort of patients with more samples will be necessary to address this limitation and develop predictive models that can be generalized, with the purpose of classifying samples with robust accuracy levels.

Due to the complexity of metabolic pathways and current limitations of our collective knowledge as represented in the KEGG database, we expect our proposed simplified cross-pathway interactions (Fig. 4) to evolve as more information is uncovered through similar studies.

## Methods

The observational study to assess the response of children with ASD to physician-directed MC treatment using saliva metabolomics and behavioral rating scales was conducted in 2020–2021. The study protocols were reviewed and approved by Ethical and Independent Review Services, an Association for the Accreditation of Human Research Protection Programs, Inc. (AAHRPP) certified institutional review board (ref 20114-01X). We confirm all methods were carried out in accordance with relevant guidelines and regulations. Parents/guardians of participating children signed an informed consent form and TD children from the control group signed an assent form. Participants, study design, data acquisition, and parental behavior assessment, were described in detail in Siani-Rose et al.^14^ and Siani-Rose et al.^15^, and briefly below.

### Participants

ASD group participants (n = 15), average age 9.4 years, male:female ratio 8:1, were recruited through CannaCenters Wellness and Education (Lawndale, CA) and Whole Plant Access for Autism (WPA4A, a 501c3 nonprofit company, Canyon Lake, CA). The inclusion criteria were: (1) ASD diagnosed by a qualified health care professional; (2) MC treatment under physician supervision for at least a year; (3) age between 6 and 12; and (4) ability to donate up to four saliva samples (0.5 ml each) using the passive drool method without discomfort. The exclusion criteria were: (1) children who require cannabis more frequently than every 8 h; (2) traumatic brain injury with any known cognitive consequence or loss of consciousness for more than 5 min; and (3) diagnosed with epilepsy.

TD group participants (n = 10), average age 9.3 years, male:female ratio 9:1, were recruited through a San Francisco online parent group, and the inclusion criteria were as follows: (1) no special education needs and (2) no individual or immediate family member diagnosed with developmental disabilities.

### Study design

All the participants in the TD group provided samples at the timepoints: PRE—morning, before MC treatment; and PEAK—when treatment was considered by parents to reach maximal impact based on their observations before the study, about 90 min after MC treatment. Some of the ASD group participants also provided samples at timepoints Post-1 and Post-2, about 180 and 270 min after MC treatment, respectively. The TD control group provided one saliva sample in the morning.

To ensure high reproducibility of the outcomes, the study was conducted as follows: (1) ASD group participants were not treated with MC for at least 8 h before PRE (washout period); (2) all participants did not consume high sugar, acid and caffeine content 1 h before any saliva sample collection; (3) all participants rinsed their mouth 20 min before saliva collection; (4) parents of all participants completed brief behavioral Likert scale 10 min before each saliva sample collection; and (5) all saliva samples were collected using the Passive Drool Collection Kit (Salimetrics, Carlsbad, CA), as previously described in detail in Siani-Rose et al.^14^.

### Untargeted metabolomic analysis

Immediately after collection all saliva samples were temporarily stored (up to 24 h) at – 20 °C, and then transferred to – 80 °C until the capillary electrophoresistime-of-flight-mass spectrometry (CE-TOF–MS) and rapid resolution liquid chromatography-time-of-flightmass spectrometry (RRLC–TOF–MS) analysis performed by Human Metabolome Technologies, Inc. (HMT, Tsuruoka, Japan) and processed as previously described in detail in Siani-Rose et al.^14^.

### Data source

Sample preparation, metabolite detection and identification, and quality control analysis were previously published in Siani-Rose et al.^14^. The metabolomics data consists of compounds detected in 40 saliva samples collected from: 15 children with ASD (15 samples at PRE, 15 matching samples at PEAK), and 10 samples from the TD control group.

### Data pre-processing

In order to adapt the data to downstream ML analysis, the following preprocessing was applied:

(1) Each non-detected compound entry in a sample was replaced by 0. This allowed the use of algorithms that cannot process missing values, while still using all of the dataset; a significant part of the dataset presents such non-detected compound entries for one or more children. A non-detected compound indicates presence in a sample below the detection level, and therefore replacing it with 0 does not fundamentally change the results we expect to obtain from our analysis. (2) Values were normalized for each individual compound across the dataset in a range (0, 1).

### Dataset preparation

Four different datasets were composed for the various ML analyses: (1) Full—a dataset consisting of all 40 samples; (2) ASD—a dataset consisting of only the 30 samples collected from children with ASD; (3) ASD PRE and ASD PEAK—a dataset of 15 entries consisting of the concatenation of the PRE data and a per-compound difference between PRE data and PEAK, referred to as the PRE + PEAK merged data. This dataset contains 2 × 645 = 1290 features (or molecules) per sample; and (4) ASD PRE/ASD PEAK—a dataset of 15 entries consisting of a per-compound difference between PRE data and PEAK data for each child in the ASD group.

The numerical datasets were completed with the addition of supplemental survey behavior data. This post-treatment survey data was converted into three outcome categories: (1) improved behavior, (2) partially improved behavior, and (3) worsening behavior. This survey data is used along with prior knowledge regarding which samples belong to children in the TD or ASD PRE group to create the prediction targets for training the ML models (Table 1). The data also included the concentrations of active cannabinoids in the treatment of each child in the ASD group in the form of numerical scales (Table 1) for each of the PEAK samples.

### ML prediction tasks

The prediction tasks described below were designed to assess the predictability of the category of a sample from the dataset, as well as to identify potential candidate compounds of interest that enable those predictions.

The full dataset was applied for training models for the ML tasks of classifying samples as TD, ASD PRE, or one of the three ASD PEAK outcome categories (i.e., improved behavior, partially improved behavior, or worsening behavior). The ASD dataset was applied for training models for the ML tasks of classifying samples as ASD PRE or as one of the three ASD PEAK outcome categories. The ASD PRE and ASD PEAK merged dataset was applied for training models for the ML tasks of classifying atypical samples in one of the three ASD PEAK outcome categories. ASD PRE/ASD PEAK dataset was applied for training models for the ML tasks of classifying ASD samples according to the active cannabinoids present in the treatment and their dosage, based on the treatment composition information. The predicted active cannabinoids included THC ranging from 0 to 50 mg per treatment, CBD ranging from 0 to 200 mg per treatment and CBG ranging from 0 to 50 mg per treatment. The ASD PRE and ASD PEAK dataset was also applied for training models for the same ML task.

### ML output analysis

The baseline algorithm used for the various prediction tasks described above was the Gradient Boosting^42^ implementation from Scikit-learn package. This algorithm provides, after training a model for a prediction task, a score for the usefulness of each feature of the training dataset for the prediction tasks.

The limited number of samples (ASD or TD control) in the datasets combined with the much larger number of features (molecules) was not optimal for training, and then run validation and testing of the prediction models. Therefore, the relevance of the models trained on each task for future prediction is limited, as the models trained most likely overfit the dataset (F1-score equal to 1 for all models trained). A future study to generate a larger dataset with many more samples will be necessary to address this limitation and develop models with the purpose of classifying samples with robust accuracy levels. However, the models trained on the present dataset can provide insights on the high dimensional data, and identify specific metabolites (out of the 645) significant for prediction and information regarding the pathways involved. Therefore, we focused on the most significant metabolites identified for each of the prediction scenarios described above.

Importance ranking (Gini importance) is computed as the normalized total reduction of the criterion brought by that feature. This feature importance ranking represents the contribution of each feature to improving the predictive ability of the model through building the model’s boosted decision tree. Though it reflects the importance of features in the final model, it is impossible to evaluate the relationship between the feature and the model predictions. Feature importance has been previously used successfully in the medical research context when using Gradient Boosting-based machine learning predictors. Previously reported applications include intensive care unit patient outcome prediction, where feature importance enabled the presentation of the top ranked features to clinicians to compare relevance of different models in and further characterize the performance of the prediction models^43^, as well as acute coronary syndrome risk prediction, to perform feature selection for machine learning model training through an iterative process to select the optimal set of features^44^.

In this study, features corresponding to the 645 distinct metabolites (identified by CE–TOF–MS and RRLC–TOF–MS in saliva) were evaluated for all samples. Out of these features, the top 50 ranking features in importance for each prediction task (top 7.7% of all available features) were considered for identifying compounds of interest.

### Ethics declarations

This study was approved by the Ethical and Independent Review Services (E&I; Lees Summit, MO), the methods were carried out in accordance with the relevant guidelines and regulations of human subjects research. Informed consent was obtained from parents/guardians of all participating children, and assent was obtained from typically-developing children.

## Data Availability

The datasets used and/or analyzed in this study are available from the corresponding author upon reasonable request.
